# TACSTD2 in gelatinous drop-like corneal dystrophy: variant functional analysis and expression in the cornea after limbal stem cell transplantation

**DOI:** 10.1038/s41439-024-00284-x

**Published:** 2024-07-16

**Authors:** Liubov O. Skorodumova, Ekaterina N. Grafskaia, Daria D. Kharlampieva, Dmitry I. Maltsev, Tatiana V. Petrova, Alexandra V. Kanygina, Elena V. Fedoseeva, Pavel V. Makarov, Boris E. Malyugin

**Affiliations:** 1 Laboratory of Human Molecular Genetics, Lopukhin Federal Research and Clinical Center of Physical-Chemical Medicine of Federal Medical Biological Agency, Moscow, Russian Federation; 2 Laboratory of Genetic Engineering, Lopukhin Federal Research and Clinical Center of Physical-Chemical Medicine of Federal Medical Biological Agency, Moscow, Russian Federation; 3 Laboratory of Neurotechnology, Federal Center of Brain Research and Neurotechnologies, Federal Medical Biological Agency, Moscow, Russian Federation; 4 Department of Trauma and Reconstructive Surgery, Helmholtz National Medical Research Center of Eye Diseases, Moscow, Russian Federation; 5 Department of Anterior Segment Transplant and Optical Reconstructive Surgery, S. Fyodorov Eye Microsurgery Complex Federal State Institution, Moscow, Russian Federation; 6 Department of Ophthalmology, A. Yevdokimov Moscow University of Medicine and Dentistry, Moscow, Russian Federation

**Keywords:** Medical genetics, Diseases, Mutation, Transcriptomics, Next-generation sequencing

## Abstract

Gelatinous drop-like corneal dystrophy (GDLD) is a rare autosomal recessive eye disease. GDLD is characterized by the loss of barrier function in corneal epithelial cells (CECs) and amyloid deposition due to pathogenic variants in the *TACSTD2* gene. Limbal stem cell transplantation (LSCT) has been suggested as an effective therapeutic alternative for patients with GDLD. However, despite LSCT, amyloid deposition recurs in some patients. The pathogenesis of recurrence is poorly studied. We present the case of a patient with GDLD. Genetic analysis revealed a homozygous deletion, NM_002353.3:c.653del, in the *TACSTD2* gene. Functional analysis in a cell model system revealed the loss of the transmembrane domain and subcellular protein mislocalization. The patient with GDLD underwent direct allogeneic LSCT with epithelial debridement followed by deep anterior lamellar keratoplasty 10 months later due to amyloid deposition and deterioration of vision. Taken together, the results of transcriptome analysis and immunofluorescence staining of post-LSCT corneal sample with amyloid deposits obtained during keratoplasty demonstrated complete restoration of wild-type TACSTD2 expression, indicating that donor CECs replaced host CECs. Our study provides experimental evidence that amyloid deposition can recur after LSCT despite complete restoration of wild-type TACSTD2 expression.

## Recurrent amyloid deposition post-LSCT: TACSTD2 gene deletion case study

Gelatinous drop-like corneal dystrophy is a rare eye disease passed down through families, causing vision loss due to a protein called amyloid building up in the cornea, the clear front surface of the eye. This study looks into the genes related to GDLD, focusing on a specific gene variant in TACSTD2, which is involved in the disease. Through sequencing of a patient’s DNA, the team found a variant in TACSTD2 linked to the protein buildup in GDLD. Results showed that despite successful LSCT, the protein built up again, indicating that replacing damaged cells with healthy ones might not prevent recurrence. The study concludes that the TACSTD2 variant contributes to GDLD by disrupting normal protein function, leading to protein buildup. This insight improves our understanding of GDLD and suggests that future treatments need to address the genetic causes. This summary was initially drafted using artificial intelligence, then revised and fact-checked by the author.

## Introduction

Gelatinous drop-like corneal dystrophy (GDLD) is a variant of epithelial and subepithelial corneal dystrophy and is a rare inherited disease characterized by bilateral amyloid mass deposition in the subepithelial area of the cornea^1,2^. The initial manifestations of the disease, such as corneal opacity, progressive vision loss, and photophobia, occur in the first two decades of life^1^.

GDLD is associated with pathogenic variants in tumor-associated calcium signal transducer 2 (*TACSTD2*)^3–5^. Also known as trophoblast antigen 2 (Trop2), TACSTD2 is a transmembrane glycoprotein encoded by the *TACSTD2* gene. It is a calcium signal transducer that is commonly expressed in the normal squamous epithelia of many organs, including the cornea. The proposed pathogenic mechanism of *TACSTD2* variants is that the mutant protein compromises tight junction formation, which is crucial for maintaining normal physiological barrier function of epithelial cells^1,6–8^. Lactoferrin is the major component of amyloid^9^. Tears are thought to be the main source of lactoferrin, which can be deposited as amyloid. Tear lactoferrin disperses through the corneal epithelium into the stroma and aggregates as an amyloid mass^7,9^.

Penetrating or deep anterior lamellar keratoplasty (DALK) and Boston type I keratoprosthesis are the predominant treatment options for severe GDLD^10–12^. However, amyloid deposits recur after keratoplasty or prosthetics^10,12^. Allogeneic limbal stem cell transplantation (LSCT) combined with penetrating or lamellar keratoplasty or superficial keratectomy has been suggested as an effective therapeutic alternative for patients with GDLD in several reports^13–17^. One report showed that LSCT performed in a patient with GDLD resulted in the replacement of host CECs with donor CECs and improved long-term survival of CECs^18^. However, in other studies, the redeposition of amyloid after LSCT was observed^13,15^.

Although the recurrence of amyloid deposition is one of the major challenges in the treatment of GLDL, the pathogenesis of this phenomenon remains unclear. Amyloid redeposition has only been studied by histologic analysis of explanted corneal grafts^11,13,19^. For example, no experimental data is available regarding whether amyloid redeposition occurs when host CECs are incompletely replaced by donor CECs after LSCT.

Here, we report the functional analysis of the *TACSTD2* frameshift variant in a patient with GDLD. The variant was identified by whole-exome sequencing (WES) and confirmed by Sanger sequencing. We analyzed the presence of wild-type *TACSTD2* at the RNA level in post-LSCT corneal samples after amyloid redeposition and examined the protein expression of TACSTD2 using IF staining. Our study of post-LSCT sample provides insight into the factors that contribute to the recurrence of amyloid deposition in grafts in patients with GDLD.

## Materials and methods

### Ethical statement

This study was approved by the Institutional Review Boards of the Helmholtz National Medical Research Center of Eye Diseases, the S. Fyodorov Eye Microsurgery Complex Federal State Institution, and the Lopukhin Federal Research and Clinical Center of Physical-Chemical Medicine of Federal Medical Biological Agency. The study was conducted in accordance with the tenets of the Declaration of Helsinki.

### Study participants

**Fig. 1:**
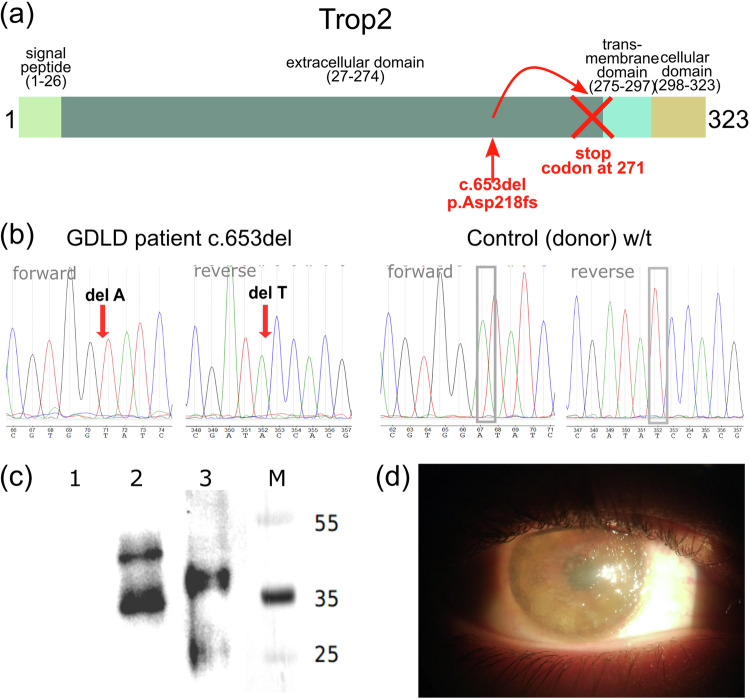
TACSTD2 (Trop2) domain structure, Sanger sequencing chromatograms of the locus of interest, Western blot analysis results, and GDLD patient right eye before DALK. **a** NM_002353.3:c.653del (NP_002344.2:p.Asp218fs) variant localized in the Trop2 extracellular domain leads to the generation of a stop codon upstream of the transmembrane domain; **b** Sanger sequencing chromatograms of the NM_002353.3:c.653del variant in the *TACSTD2* gene. Arrows indicate deleted nucleotides. **c** Western blot analysis of the Trop2 protein. (1) Expi293F cells; (2) Expi293F cells transfected with a plasmid encoding w/t Trop2 (pHum-Ref); (3) Expi293F cells transfected with a plasmid encoding Trop2 with an Asp218fs mutation (pHum-Gdld); M molecular weight marker, kDa; (**d**) Slit lamp image of the right eye of a patient with GDLD prior to DALK.

The patient was a male who was clinically diagnosed with GDLD at the age of 15 years. The diagnosis was based on the following characteristic symptoms: grayish and yellow deposits under the epithelium and in the corneal stroma, vascularization, bilateral corneal lesions and decreased visual acuity. The deposits had a typical mulberry appearance (Fig. 1d). The patient had been followed at the Helmholtz National Medical Research Center of Eye Diseases since the age of 19 years and underwent DALK of the left eye at age 20 years. One year later, due to limbal stem cell deficiency (LSCD) in the right eye, direct allogeneic LSCT was performed. At the age of 22 years, he was asked to participate in a molecular research study. He agreed and provided written informed consent. A blood sample was collected for genetic analysis. Ten months after LSCT, the patient underwent DALK surgery on his right eye. During keratoplasty, a sample of the cornea from the right eye was collected.

The control cornea for IF staining was obtained from a donor eye of a 45 year-old male at the S. Fyodorov Eye Microsurgery Complex Federal State Institution Eye Bank.

### Direct allogeneic LSCT

Direct allogeneic LSCT with epithelial debridement was performed on the right eye of the GDLD patient. A donor corneoscleral disc was obtained from a cadaver eye prior to surgery. The graft was cut with a blade to 1⁄4 of the full thickness of the cornea and sclera. The graft consisted of 1−1.5 mm of cornea, 2−2.5 mm of sclera, and 3−4 mm of full-thickness limbal conjunctiva. The patient’s corneal epithelium was removed, leaving Bowman’s membrane intact. The patient then underwent circular limbal peritomy and excision of the perilimbal and limbal tissues. The donor corneoscleral graft was placed on the patient’s limbal area and secured with 10–0 nylon sutures to the cornea (inner edge of the graft), sclera (outer scleral edge of the graft), and the patient’s conjunctiva edge-to-edge, including the episclera. At the end of the surgery, the amniotic membrane was placed over the entire cornea and secured with sutures, and a soft contact lens was placed. Dexamethasone subconjunctival injections (2.5 mg) were administered in the early postoperative period for 7 days, followed by a 1.0 ml parabulbar injection of diprospan (a combination of betamethasone sodium phosphate and betamethasone dipropionate combination). Dexamethasone (0.1%) was also administered three times a day for 1 year. Immunosuppressive therapy consisted of oral administration of cyclosporine A (100 mg twice a day for several years to date). The soft contact lens was removed 3 weeks after amniotic membrane lysis.

### Corneal sample conservation and nucleic acid extraction

After excision, cornea samples from the GDLD patient and donor were immediately dissected into two equal semicircles: one half was immersed in RNAlater solution (Thermo Fisher Scientific, Waltham, Massachusetts, USA) for RNA and DNA stabilization, and the other half of the cornea was preserved in 10% neutral buffered histological formalin and embedded in a paraffin block. DNA was extracted from the patient’s blood and the control cornea. RNA extraction was performed on half of the patient’s cornea preserved in RNAlater solution. The cornea was homogenized using the TissueLyser (Qiagen, Hilden, Germany) with stainless steel beads.

### Exome sequencing and bioinformatic analysis

The GDLD patient exome library was constructed using the SureSelectXT2 Target Enrichment System for the Illumina platform (Agilent, Santa Clara, California, USA) and SureSelect Human All Exon V7 probes (Agilent, Santa Clara, California, USA) according to the manufacturer’s instructions. The resulting paired-end libraries were sequenced on an Illumina NovaSeq 6000 instrument (Illumina, San Diego, California, USA) using 2 × 100 cycles. Whole exome sequencing data were uploaded to the NCBI Sequence Read Archive under accession number PRJNA714952.

Primary quality control of the raw sequencing reads was performed using FastQC^20^. Reads were aligned to the reference human genome (build GRCh38) using BWA-MEM 0.7.17 [Aligning sequence reads, clone sequences and assembly contigs with BWA-MEM], and duplicates were marked using MarkDuplicates from Picard 2.23.1 [https://broadinstitute.github.io/picard/]. Variant calling was performed with DeepVariant 1.4.0 using the model for Illumina Whole Exome Sequencing (model_type WES)^21^. Calling was restricted to the target regions of the SureSelect Human All Exon V7 Kit provided by Agilent Technologies. Variant annotation was performed using ANNOVAR^22^.

### TACSTD2 variant analysis

To validate the variant in the *TACSTD2* gene, PCR was performed using the following primers: forward 5’-GCTGCACCCCAAGTTCGT-3’ and reverse 5’-GCTGCACCCCAAGTTCGT-3’. The amplicons were subjected to Sanger sequencing.

### Cloning and transient transfection

Expi293F cells (Thermo Fisher Scientific, Waltham, Massachusetts, USA) were cultured in DMEM supplemented with 10% inactivated FBS and 10 µg/ml gentamicin. The pHum-Ref and pHum-Gdld plasmids were generated from a pcDNA3.4 plasmid (Thermo Fisher Scientific, Waltham, Massachusetts, USA). The fragments encoding wild-type and mutant Trop2 were amplified using the primers 5’-agtcggatccaccatggctcggggccccggcct-3’ and 5’-agtcctcgagctacaagctcggttcctttctcaac-3’ and DNA with a reference sequence (Supplementary Fig. 1) or GDLD patient DNA, respectively, and cloned at the BamHI/XhoI sites. The nucleotide sequences of the cloned fragments were verified by Sanger sequencing. To obtain wild-type and mutant Trop2-producing cells, Expi293F cells were transfected with the constructed vectors using Lipofectamine 3000 Transfection Reagent (Thermo Fisher Scientific, Waltham, Massachusetts, USA) according to the manufacturer’s instructions.

### Western blotting analysis

Western blot analysis was performed on Day 2 after transfection. The cells were washed with HBSS, centrifuged at 3000 × g for 10 min and resuspended in 100 µl of HBSS. The cell suspension was then sonicated for 30 s and centrifuged at 14,000 × g for 5 min, after which the supernatant was collected. Equal aliquots of supernatants from each sample were analyzed by Western blotting to detect the presence of Trop2. Samples diluted in Laemmli sample buffer were heated to 95 °C for 5 min. The probes were separated by 13.5% SDS-PAGE. After separation, the proteins were transferred (semidry transfer, 1 mA/cm2 for 1 h) to a Hybond-P PVDF membrane in Tris-glycine transfer buffer (48 mM Tris, 39 mM glycine, 0.04% SDS, 20% methanol). The membrane was blocked with 3% Blotting Grade Blocker Nonfat Dry Milk in PBST and incubated with primary anti-Trop2 antibodies (rabbit polyclonal PA5-13638, Thermo Fisher Scientific, Waltham, Massachusetts, USA) at a 1:1000 dilution at 4°C overnight. The membrane was washed three times with PBST, incubated with secondary HRP-conjugated donkey anti-rabbit IgG antibody (NA934; GE Healthcare, Piscataway, New Jersey, USA) at a 1:10000 dilution for 1 h, and then washed three times. The target protein was visualized using ECL Plus Western blotting detection reagents (GE Healthcare, Little Chalfont, United Kingdom). Signals were detected using a ChemiDoc XRS+ system (Bio-Rad Laboratories, Hercules, California, USA).

### Immunofluorescence staining of the Expi293F cells

For IF staining on Day 2 after transfection, the cells were fixed with methanol for 20 min at +4 °C. After blocking, the cells were incubated with primary anti-Trop2 antibodies (rabbit polyclonal PA5-13638 X; Thermo Fisher Scientific, Waltham, Massachusetts, USA) overnight at +4 °C. Goat anti-rabbit IgG Alexa Fluor 594-conjugated secondary antibodies (ab150084; Abcam, Cambridge, United Kingdom) were applied for 1 h at RT (room temperature). Cell nuclei were stained with Hoechst. The endoplasmic reticulum (EPR) was stained with the SelectFX Alexa Fluor 488 Endoplasmic Reticulum Labeling Kit (Thermo Fisher Scientific, Waltham, Massachusetts, USA). Trop2 protein localization was assessed using a Nikon Eclipse Ti-E confocal microscope equipped with a Nikon A1 LFOV camera and a Plan Apo *λ* 100 x NA 1.40 oil immersion objective. Lasers at 405 nm, 488 nm, and 561 nm were used for fluorescence detection.

### Transcriptome library construction and sequencing

The cornea RNA of the patient was used for transcriptome library construction. For this purpose, the NEBNext Ultra II Directional Library Prep Kit for Illumina (New England Biolabs, Ipswich, Massachusetts, USA) and Multiplex Oligos for Illumina (96 Indices) (New England Biolabs, Ipswich, Massachusetts, USA) were utilized. Ribosomal RNA was depleted using the NEBNext rRNA Depletion Kit (Human/Mouse/Rat) (New England Biolabs, Ipswich, Massachusetts, USA). The library was sequenced on the Illumina HiSeq 2500 instrument with 2 × 125 cycles using the HiSeq SBS Kit v4 (Illumina, San Diego, California, USA). RNA-Seq data were uploaded to the NCBI Sequence Read Archive under accession number PRJNA714952.

### Immunofluorescence staining of the cornea

Immunofluorescence staining was performed according to the Abcam IHC staining protocol for paraffin sections and fluorescence detection. Briefly, GDLD and control formalin-fixed corneas were processed and embedded in paraffin blocks. Tissue sections were mounted on poly-L-lysine-coated slides and processed for deparaffinization and rehydration, followed by antigen retrieval. The sections were permeabilized with 0.15% Triton X-100 in PBS for 5 min. The sections were blocked for 1 h with 3% BSA at room temperature. The corneal sections were then incubated overnight at +4 °C with primary anti-Trop2 (rabbit polyclonal PA5-13638; Thermo Fisher Scientific, Waltham, Massachusetts, USA) or anti-Keratin 12/K12 (rabbit monoclonal ab185627; Abcam, Cambridge, United Kingdom) antibodies at a 1:50 dilution. The next day, the tissue sections were washed with PBS and stained with Alexa Fluor 594-conjugated secondary goat anti-rabbit IgG antibodies (ab150084; Abcam, Cambridge, United Kingdom) for 1 h at room temperature (1:500 dilution). Hoechst 33342 was used to visualize the nuclei. Images were acquired after mounting with antifade fluorescence mounting medium using a Nikon Eclipse Ni-E fluorescence microscope with a 63× oil objective.

## Results

### Whole exome sequencing and identification of a pathogenic variant

We analyzed germline variants in the whole exome sequencing data of the GDLD patient. A homozygous deletion, NM_002353.3:c.653del (rs780819073), which leads to a frameshift (NP_002344.2:p.Asp218fs) and generation of a premature stop codon (Fig. 1a) was identified. Sanger sequencing of this locus in the GDLD patient validated the revealed frameshift variant. The control subject had a reference sequence at this locus (Fig. 1b). Because the patient with GDLD did not accept contact with relatives, we were unable to perform segregation analysis or obtain information about consanguineous marriages in the family.

### Functional analysis of the NM_002353.3:c.653del variant in a cell model

We used a transient transfection cell model to evaluate the effect of the NM_002353.3:c.653del variant on expression at the protein level. Expi293F cells were transfected with vectors encoding wild-type or mutant Trop2 (pHum-Ref and pHum-Gdld plasmids, respectively).

The protein expression of Trop2 was analyzed by immunoblotting (Fig. 1c). No Trop2 signal was detected in intact Expi293F cells (Lane 1), which served as a negative control for antibody specificity. Western blot analysis revealed two bands in Lanes 2 and 3, possibly representing different glycosylation states of Trop2, as previously reported^23,24^. The heaviest product in cells transfected with the plasmid encoding wild-type Trop2 was ~50 kDa (Lane 2), the predicted molecular weight of glycosylated Trop2. The heaviest product synthesized in cells transfected with a plasmid encoding mutant Trop2 was ~40 kDa (Lane 3). Thus, the Trop2 products produced by cells expressing mutant Trop2 were lower in molecular weight than those produced by cells expressing wild-type Trop2, as shown by the blot.

**Fig. 2:**
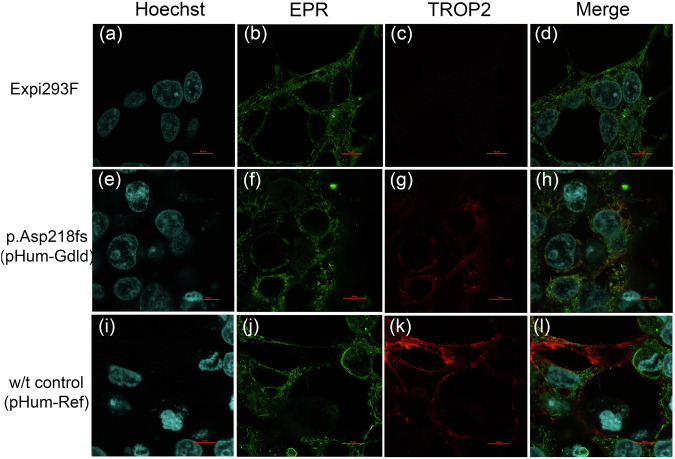
Subcellular localization of the wild-type or NP_002344.2:p.Asp218fs mutant Trop2 protein. Immunofluorescence staining of intact Expi293F cells is presented in (**a**–**d**). Expi293F cells transfected with a plasmid encoding Trop2 with the Asp218fs mutation (pHum-Gdld) are presented in (**e**–**h**). Expi293F cells transfected with plasmids encoding w/t Trop2 (pHum-Ref) are presented in (**i**–**l**). The green color indicates EPR (**b**, **f**, **j**); the red color indicates Trop2 (**c**, **g**, **k**). Scale bars: 10 μm.

According to the described domain structure of Trop2, the premature stop codon resulting from the c.653del variant is located upstream of the locus encoding the transmembrane domain (Fig. 1a)^25^. Loss of the transmembrane domain could lead to mislocalization of the mutant Trop2 protein. Therefore, we examined the intracellular localization of the wild-type and mutant Trop2 proteins by IF staining of transfected cells (Fig. 2).

Immunofluorescence staining revealed a dramatic difference in the subcellular localization of Trop2. In the cell model system, wild-type Trop2 localized to the cell membrane as it did in the corneal epithelium (Fig. 2k, l). Mutant Trop2 localized intracellularly (Fig. 2g, h).

### Clinical outcome of LSCT and wild-type TACSTD2 RNA and protein expression in a post-LSCT corneal sample

**Fig. 3:**
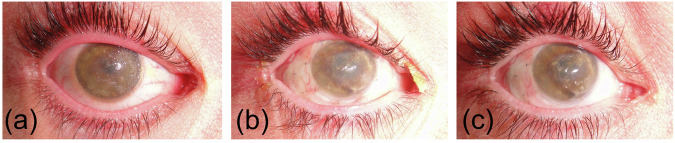
Photographs of the right eye of a patient with GDLD. (**a**) before LSCT; (**b**) 2.5 months after LSCT; (**c**) 6 months after LSCT.

The GDLD patient underwent circular LSCT with epithelial debridement of the right eye (Fig. 3b). The corneal surface became smooth without mulberry-like deposits. At the time of LSCT, his visual acuity in the right eye was 0.01. After LSCT, the patient experienced relief of subjective symptoms, including photophobia and tearing. Six months after LSCT, amyloid deposits were again visible in the right eye (Fig. 3c). Ten months after LSCT, the patient with GDLD underwent DALK in the right eye. Before keratoplasty, he could only count fingers with his right eye (Fig. 1d). Thus, GDLD progressed during this period despite LSCT. Four months after DALK, the visual acuity of the right eye reached its maximum value of 0.3.

**Fig. 4:**
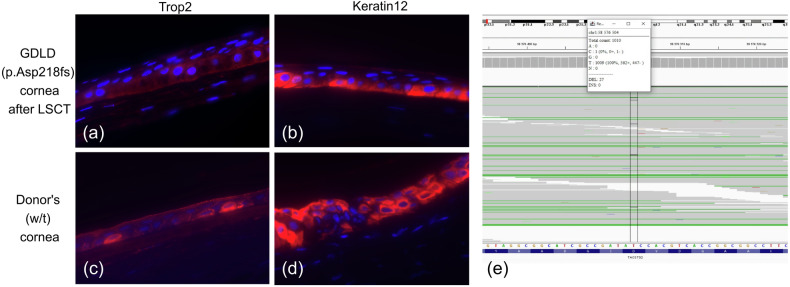
TACSTD2 expression at the protein and RNA levels. **a**–**d** Expression of the epithelial markers Trop2 and cytokeratin 12 in corneal samples from GDLD patient and control donor. Immunofluorescence staining of the GDLD patient cornea (p.Asp218fs) after LSCT is shown in (**a**, **b**). Immunofluorescence staining of the control donor cornea (w/t) is shown in (**c**, **d**). Red indicates Trop2 (**a** and **c**) or keratin 12 (**b** and **d**). **e** *TACSTD2* expression in GDLD patient cornea after LSCT (screenshot of the IGV browser interface showing coverage at the NM_002353.3:c.653del variant site; indicated by black vertical lines).

To determine whether amyloid deposition recurs after LSCT due to incomplete replacement of host CECs by donor CECs, we analyzed the expression of wild-type TACSTD2 at the RNA and protein levels in the post-LSCT corneal sample with amyloid deposits obtained during keratoplasty. One half of the patient’s post-LSCT cornea was subjected to transcriptome sequencing, while the other half was subjected to IF staining. Ninety-seven percent of the reads (983/1010) overlapping the c.653del variant site harbored a reference nucleotide (Fig. 4e). Thus, RNA from corneal sample was predominantly synthesized by donor CECs harboring wild-type *TACSTD2*.

Immunofluorescence staining of the GDLD patient post-LSCT cornea and the control donor cornea revealed positivity for the Trop2 (Fig. 4a, c) and Keratin 12 proteins (Fig. 4b, d). Keratin 12 was used as a surrogate marker for corneal epithelium integrity.

Thus, the combined results of variant calling from the transcriptome data and IF staining of the post-LSCT corneal sample showed that almost all TACSTD2 was wild-type, which can be explained by the complete replacement of host CECs by donor CECs.

## Discussion

Gelatinous drop-like corneal dystrophy is a rare type of corneal dystrophy characterized by subepithelial and stromal amyloid deposition. Tsujikawa et al. first demonstrated that loss-of-function variants in the *TACSTD2* gene are causal for GDLD^3^. Amyloid deposition has also been reported in patients with missense variants^4,26^. The reported variants have been reviewed in Nagahara et al. and Zhu et al.^5,27^. A defective Trop2 protein is responsible for the improper subcellular localization of tight junction-related proteins, which leads to the loss of CEC barrier function^7^.

Treatment options for such patients are limited and commonly include superficial keratectomy, keratoplasty with or without LSCT or the implantation of an artificial cornea device. The redeposition of amyloid in corneal grafts leads to unfavorable long-term outcomes. The results of LSCT in combination with penetrating or lamellar keratoplasty or superficial keratectomy in patients with GDLD have been described in several studies^13–17^. Shimazaki and coauthors^13^ reported 8 out of 9 eyes without recurrence after LSCT with penetrating keratoplasty (PKP) for 4 years. Movahedan et al^14^. reported similar results: 4 eyes were free of disease after PKP or superficial keratectomy for 12−36 months of follow-up. Lang and colleagues^15^ reported a median time to recurrence of 3 years in 7 eyes after LSCT performed simultaneously with PKP. Omoto et al^16^. performed keratolimbal allograft (KLAL) transplantation simultaneously with DALK in two eyes of a patient with GDLD and LSCD. Azher et al. performed KLAL simultaneously with superficial keratectomy before or after PKP in one patient with GDLD^17^. They reported clear PKP grafts after 2 and 3 years of follow-up. Combined, the data from these reports revealed a 25% recurrence rate with a median follow-up of 50 months (Supplementary Table 1)^13–17^. However, control groups were not included in the published studies; therefore, definitive conclusions about the increase in recurrence-free survival after keratoplasty or keratectomy combined with LSCT compared to that in conventional keratoplasty cannot yet be drawn.

In addition, after LSCT, cells of different genetic origins interact in the epithelium of the host and at least one donor. This provides an option to assess the degree of replacement of mutated host CECs by nonmutated donor CECs. This may help to better understand the relationship between the presence of functional TACSTD2 and amyloid redeposition.

A homozygous deletion, NM_002353.3:c.653del, in the *TACSTD2* gene was detected in a Russian patient with GDLD. The allele frequency of this variant is extremely rare: 1.22e-5 (3/245036, GnomAD_exome, 0 homozygotes). Previously, Markoff A. and coauthors reported this variant in Turkish sisters with GDLD^28^. However, this variant has not been annotated in the ClinVar database until now (SCV002556359). We performed a functional analysis of the NM_002353.3:c.653del variant in a cell model. According to information on the Trop2 domain structure, NM_002353.3:c.653del leads to the generation of a premature stop codon immediately upstream of the transmembrane domain, which is required for Trop2 localization to the membrane^25^. Immunofluorescence staining of the transfected cells revealed that mutant Trop2 was localized intracellularly. These results are in good agreement with the prediction of the loss of the transmembrane domain and confirm the deleterious effect of the NP_002344.2:p.Asp218fs variant.

The patient underwent LSCT with epithelial debridement of the right eye. Deposits were not visible immediately after LSCT (they were removed with the epithelium). Bowman’s membrane was protected from tear lactoferrin deposition by the amniotic membrane for 3 weeks, while donor CECs repopulated the epithelial layer. Nevertheless, amyloid deposition recurred 6 months later. In our case, the time to recurrence was shorter than that of LSCT accompanied by PKP (median 3 years)^15^. Thus, our findings suggest that LSCT combined with corneal epithelial debridement was less effective at preventing amyloid deposition than LSCT combined with PKP. Furthermore, our study provides experimental evidence that amyloid deposition can recur after LSCT despite the complete replacement of host CECs by donor CECs, as demonstrated by the combined results of IF staining and transcriptome analysis of the post-LSCT corneal sample, which clearly revealed almost exclusive wild-type TACSTD2 expression.

The presence of other pathways through which lactoferrin, the principal component of amyloid, enters the cornea may explain why restoration of TACSTD2 function in the epithelium is not sufficient to prevent amyloid deposition. Initially, lactoferrin was identified in mammalian milk^29^. However, subsequent studies have demonstrated its presence in blood plasma and a range of exocrine secretions, including tears, nasal exudates, saliva, and others. Plasma lactoferrin is predominantly derived from neutrophils^30^. Tear lactoferrin is secreted by acinar epithelial cells of lacrimal tissue^31^. Therefore, other potential routes for lactoferrin to enter the cornea may include lacrimal fluid perfusion through the conjunctiva and via the bloodstream in cases of corneal neovascularization. The concentration of lactoferrin in tears is ~1000 times greater than that in plasma (0.41 − 13.5 mg/mL in tears vs. 0.2 − 1.5 μg/mL in plasma)^32,33^. Most likely, LSCT or keratoplasty cannot prevent amyloid deposition because tear lactoferrin can be perfused through conjunctival epithelial cells. It has been shown that conjunctival epithelial cells harboring the homozygous p.Gln118fs variant do not synthesize the Trop2 protein^34^. Thus, the conjunctival epithelium is as permeable as the cornea. It is possible that lactoferrin diffuses into the sclera and then reaches the corneal stroma, where it is deposited as amyloid. This hypothesis needs to be investigated experimentally.

The experimental study of amyloid deposition is complicated by the fact that although there is a mouse model of GDLD, neither *Tacstd2* + */+* nor *Tacstd2* − /− aged mice showed Congo red–positive amyloid staining. This may be explained by the low lactoferrin concentration in mouse tears (0.2 µg/ml)^35,36^.

In conclusion, we confirmed the pathogenicity of the homozygous *TACSTD2* gene variant NM_002353.3:c.653del in a patient with GDLD. Our functional study showed that this frameshift variant leads to loss of the transmembrane domain and aberrant subcellular localization of the protein. We showed that LSCT combined with epithelial debridement was less effective at preventing amyloid deposition than LSCT combined with PKP. Taken together, the results of IF staining and transcriptome analysis provided experimental evidence that amyloid deposits reappeared after LSCT despite complete restoration of wild-type TACSTD2 expression in the corneal epithelium.

## Supplementary information

Supplementary Information

## Data Availability

Data describing the identified variants have been submitted to the ClinVar Database (https://www.ncbi.nlm.nih.gov/clinvar/) under accession no. SCV002556359. RNA-Seq and whole-exome sequencing data were uploaded to the NCBI Sequence Read Archive under accession no. PRJNA714952. (https://www.ncbi.nlm.nih.gov/bioproject/PRJNA714952).
