# Supplementary material for: TACSTD2 in gelatinous drop-like corneal dystrophy: variant functional analysis and expression in the cornea after limbal stem cell transplantation
Source: Hum Genome Var. 2024 Jul 16;11:26. doi: 10.1038/s41439-024-00284-x (PMC11252363; doi:10.1038/s41439-024-00284-x)
Supplement: Supplementary file 1 — Supplementary Information [file 41439_2024_284_MOESM1_ESM.docx]

**Supplementary Figure 1.** Sanger sequencing chromatograms of the NM_002353.3:c.653 in *TACSTD2* gene in the reference DNA sample used for pHum-Ref plasmid construction.


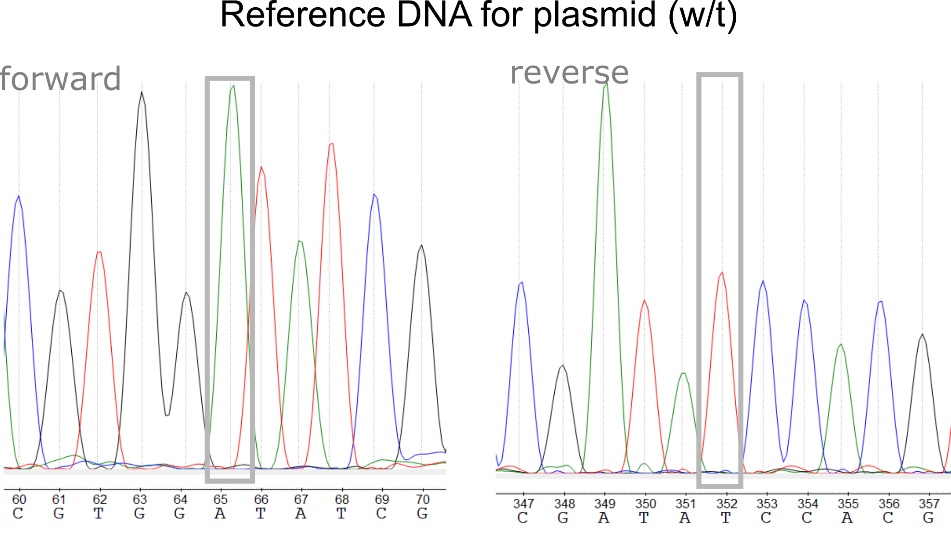


**Supplementary Table 1**. Summary data on GDLD patients’ follow-up and amyloid deposition recurrence after LSCT combined with penetrating or lamellar keratoplasty or superficial keratectomy.

| Reference | Participant # | Follow-up, mo | Recurrence |
| --- | --- | --- | --- |
| Shimazaki et al. (13) | 1 | 79,7 | no |
|  | 2 | 11,7 | no |
|  | 3 | 22,5 | no |
|  | 4 | 81 | no |
|  | 5L | 75,3 | yes |
|  | 5R | 39,6 | no |
|  | 6L | 65,9 | no |
|  | 6R | 61,1 | no |
|  | 7 | 8,9 | no |
| Movahedan et al. (14) | 1 | 36 | no |
|  | 2 | 24 | no |
|  | 3 | 12 | no |
|  | 4 | 20 | no |
| Omoto et al. (16) | 1L | 36 | no |
|  | 1R | 22 | no |
| Azher et al. (17) | 1L | 36 | no |
|  | 1R | 24 | no |
| Lang et al. (15) | 1 | 72 | yes |
|  | 2 | 72 | yes |
|  | 3 | 72 | yes |
|  | 4 | 72 | yes |
|  | 5 | 72 | yes |
|  | 6 | 72 | no |
|  | 7 | 72 | no |
| Median | | 50,35 |  |
| Recurrence rate | | | 0,25 |
